# TRAIL-R4 Promotes Tumor Growth and Resistance to Apoptosis in Cervical Carcinoma HeLa Cells through AKT

**DOI:** 10.1371/journal.pone.0019679

**Published:** 2011-05-20

**Authors:** Najoua Lalaoui, Aymeric Morlé, Delphine Mérino, Guillaume Jacquemin, Elisabetta Iessi, Alexandre Morizot, Sarah Shirley, Bruno Robert, Eric Solary, Carmen Garrido, Olivier Micheau

**Affiliations:** 1 U866, INSERM (Institut National de la Santé et de la Recherche Médicale), Dijon, France; 2 Faculté de Médecine et de Pharmacie, Université de Bourgogne, Dijon, France; 3 Institut de Recherche en Cancérologie de Montpellier (IRCM), U896, INSERM (Institut National de la Santé et de la Recherche Médicale), Montpellier, France; 4 CRLC Val d'Aurelle-Paul Lamarque, Université Montpellier1, Montpellier, France; 5 U1009, INSERM (Institut National de la Santé et de la Recherche Médicale), Villejuif, France; 6 Institut Gustave Roussy, Univ. Paris XI, Villejuif, France; 7 Centre Hospitalier Universitaire Dijon, Dijon, France; 8 Centre Georges-François Leclerc, Dijon, France; Pavillon Kirmisson, France

## Abstract

**Background:**

TRAIL/Apo2L is a pro-apoptotic ligand of the TNF family that engages the apoptotic machinery through two pro-apoptotic receptors, TRAIL-R1 and TRAIL-R2. This cell death program is tightly controlled by two antagonistic receptors, TRAIL-R3 and TRAIL-R4, both devoid of a functional death domain, an intracellular region of the receptor, required for the recruitment and the activation of initiator caspases. Upon TRAIL-binding, TRAIL-R4 forms a heteromeric complex with the agonistic receptor TRAIL-R2 leading to reduced caspase-8 activation and apoptosis.

**Methodology/Principal Findings:**

We provide evidence that TRAIL-R4 can also exhibit, in a ligand independent manner, signaling properties in the cervical carcinoma cell line HeLa, through Akt. Ectopic expression of TRAIL-R4 in HeLa cells induced morphological changes, with cell rounding, loss of adherence and markedly enhanced cell proliferation *in vitro* and tumor growth *in vivo*. Disruption of the PI3K/Akt pathway using the pharmacological inhibitor LY294002, siRNA targeting the p85 regulatory subunit of phosphatidylinositol-3 kinase, or by PTEN over-expression, partially restored TRAIL-mediated apoptosis in these cells. Moreover, the Akt inhibitor, LY294002, restituted normal cell proliferation index in HeLa cells expressing TRAIL-R4.

**Conclusions/Significance:**

Altogether, these results indicate that, besides its ability to directly inhibit TRAIL-induced cell death at the membrane, TRAIL-R4 can also trigger the activation of signaling pathways leading to cell survival and proliferation in HeLa cells. Our findings raise the possibility that TRAIL-R4 may contribute to cervical carcinogenesis.

## Introduction

TRAIL/Apo2L is regarded as a promising anticancer agent for cancer therapy and is currently being evaluated in clinical trials [1]. TRAIL binds to four membrane-anchored receptors: TRAIL-R1 (DR4), TRAIL-R2 (DR5, Killer, TRICK), TRAIL-R3 (DcR1, LIT, TRID) or TRAIL-R4 (DcR2, TRUNDD), and one soluble receptor, osteoprotegerin (OPG) [1]. TRAIL induces cell death through its interaction with either TRAIL-R1 or TRAIL-R2. These two agonistic receptors harbor, within their cytoplasmic region, a relatively small amino acid stretch called the death domain (DD), which is necessary and sufficient to transduce the death signal [2]. Activation of TRAIL-R1 or TRAIL-R2 by trimeric TRAIL induces the recruitment of the adaptor protein FADD (Fas-associated death domain protein) *via* homotypic interactions with their respective DD, allowing in turn the recruitment of the initiator caspases, procaspases-8 and -10 [3], [4], leading to the formation of the death-inducing signaling complex (DISC) [5]. Within the DISC, the initiator caspases-8 and -10 undergo catalytic cleavage inducing their release to the cytosol and the triggering of the caspase cascade that ultimately leads to apoptosis. In contrast, TRAIL binding to TRAIL-R3 or TRAIL-R4 fails to induce the apoptotic machinery because none of these receptors harbor a functional DD [6]. TRAIL-R3 is anchored to the membrane via its glycosyl-phosphatidylinositol tail (GPI), whereas TRAIL-R4 is addressed to the cell surface through a transmembrane domain but includes a truncated DD that is unable to recruit the adaptor protein FADD [7].

Expression of TRAIL-R3 or TRAIL-R4 confers resistance to TRAIL-induced cell death in several tumor cell lines and primary tumors [8], [9], [10], [11], [12], [13]. These antagonistic receptors, coined “decoy receptors”, were initially proposed to act as competitors to TRAIL-R1 and TRAIL-R2 for TRAIL binding [14]. However, we and others have provided evidence that TRAIL-R4 should rather be considered as a regulatory receptor, because TRAIL-R4 is able to interact with TRAIL-R2 within the TRAIL DISC and to impair caspase-8 activation [10], [12], [15]. In this study, we provide new evidence that TRAIL-R4 exhibits a TRAIL-independent signaling activity that gives rise to oncogenic-like properties in HeLa cells, mainly through the activation of Akt.

## Results

### TRAIL-R4 ectopic expression in HeLa cells markedly changes cell morphology, cell proliferation and tumor growth

**Figure 1:**
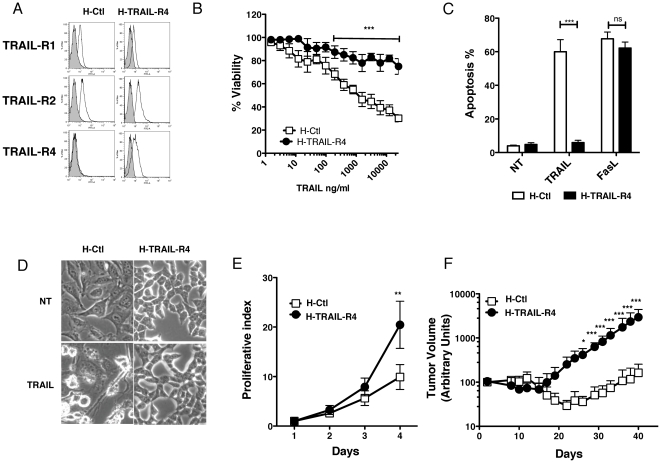
TRAIL-R4 protects HeLa cells from TRAIL-induced apoptosis and enhances tumor cell growth *in vitro and in vivo*. (A) HeLa stably transduced with retroviruses encoding TRAIL-R4 (H-TRAIL-R4), or the empty mock retroviral vector (H-Ctl), were analyzed by flow cytometry for TRAIL receptor staining as indicated. (B) Cellular viability of the populations was evaluated by PMS/MTS 24 hours after treatment with increasing concentrations of recombinant His-TRAIL. HeLa control (H-Ctl in open squares) and HeLa expressing TRAIL-R4 (H-TRAIL-R4 in filled circles). Mean viability % and SD from three independent experiments are shown (mean ± SD). ***P<0.001, two-way ANOVA with Bonferroni post-tests, H-TRAIL-R4 compared with H-Ctl-Mock. (C) Apoptosis-induced by TRAIL (500 ng/ml) in H-Ctl and H-TRAIL-R4 cells 24 hours after stimulation. Apoptosis was determined by Hoechst staining. Data are representative of at least three independent experiments. ***P<0.001, student t-test. (D) Representative light microscopic picture of H-Ctl versus H-TRAIL-R4 treated or not (NT) with 500 ng/ml TRAIL for 16 hours. (E) Cell proliferative index was followed for 4 days and measured using CFSE by flow cytometry. Mean analysis from three independent experiments is shown. HeLa control (H-Ctl in open squares) and HeLa expressing TRAIL-R4 (H-TRAIL-R4 in filled circles). (F) Time dependent growth of HeLa control (H-Ctl in open squares) and HeLa expressing TRAIL-R4 (H-TRAIL-R4 in filled circles) in nude mice after xenograft (n = 10). These results represent the mean tumor volume in arbitrary units ± SD of a representative experiment performed with six to seven mice per group. (E) and (F) *P<0.05 and ***P<0.001, two-way ANOVA with Bonferroni post-tests, H-TRAIL-R4 compared with H-Ctl-Mock.

Ectopic TRAIL-R4 expression to physiological levels in HeLa cells (Figure 1A), as well as in other tumors [15], by use of retroviral vectors, affords good selective protection against TRAIL-induced cell death, but not Fas ligand (Figure 1B and C). Strikingly, HeLa cells expressing TRAIL-R4 (H-TRAIL-R4) undergo drastic morphological changes including cell rounding and loss of adherence (Figure 1D). As compared to control cells (H-Ctl) infected with an empty vector, H-TRAIL-R4 cells exhibited a higher proliferative index (Figure 1E). This increase in cell proliferation is however most likely independent of TRAIL itself, since the recombinant fusion protein Fc-TRAIL-R2 failed to affect proliferation in H-TRAIL-R4 cells (Figure S1A). In agreement with these findings, TRAIL levels were undetectable in the supernatant or at the surface of H-TRAIL-R4 cells (not shown). The drastic changes in cell morphology and proliferative status prompted us to check whether TRAIL-R4 overexpression confers tumor growth advantage *in vivo*. Parental (H-Ctl) and TRAIL-R4 expressing cells (H-TRAIL-R4) were implanted into nude mice in the left and the right flank, respectively of the same animal (Fig. S1B) and tumor growth was followed for 32 days (Figure 1F). Remarkably, TRAIL-R4 expressing HeLa cells exhibited a clear tumor growth advantage as compared to control cells in nude mice.

**Figure 2:**
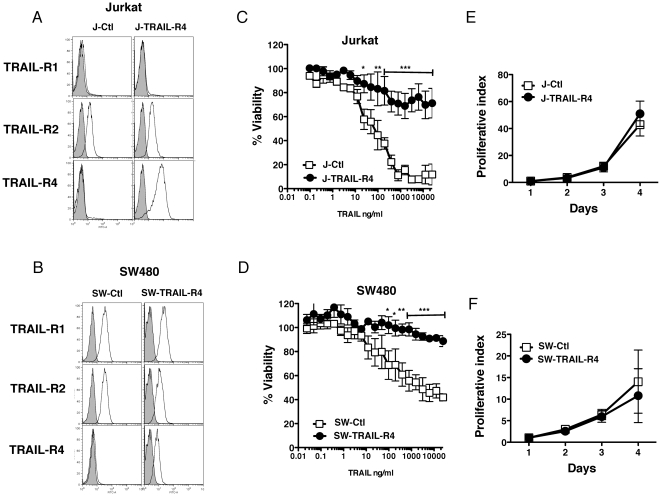
TRAIL-R4 ectopic expression fails to promote cell proliferation in Jurkat and SW480 cells. (A and B) Jurkat and SW480 cells were stably transduced with retroviruses encoding TRAIL-R4 (J-TRAIL-R4 and SW-TRAIL-R4), or the empty mock retroviral vector (J-Ctl and SW-Ctl), respectively. Cells were analyzed by flow cytometry for TRAIL receptor staining as indicated. (C and D) Cellular viability was evaluated by PMS/MTS 24 hours after treatment with increasing concentrations of recombinant His-TRAIL. Jurkat or SW480 control cells (J-Ctl or SW-Ctl in open squares) and Jurkat or SW480 expressing ectopically TRAIL-R4 (J-TRAIL-R4 or SW-TRAIL-R4 in filled circles). (E and F) Cell proliferative index was measured as in Figure 1. Mean proliferative index, viable cells and SD from three independent experiments are shown (mean ± SD). *P<0.05, **P<0.01 and ***P<0.001, two-way ANOVA with Bonferroni post-tests, J- or SW-TRAIL-R4 compared with J- and SW-Ctl-Mock respectively.

We next checked whether TRAIL-R4 ectopic expression affected cell proliferation in two other TRAIL-sensitive tumor cell lines, the Jurkat T cell lymphoma and the colon carcinoma SW480. To address this question, Jurkat and SW480 cell lines were infected with an empty vector (J-Ctl and SW-Ctl) or a retroviral vector encoding TRAIL-R4 (J-TRAIL-R4 and SW-TRAIL-R4). Expression levels of TRAIL-R4 and TRAIL-R2 were analyzed by flow cytometry (Figure 2A and B) and cell sensitivity to TRAIL-induced cell death was determined by PMS-MTS assay. Similar to HeLa cells, ectopic expression of TRAIL-R4 in Jurkat or SW480 cells inhibited TRAIL-induced cell death in a dose-dependent manner (Figure 2C and D). TRAIL-R4 expression, however, induced no particular modification of cell proliferation as compared to control cells (Figure 2E and F), and no change in cell morphology could be observed (not shown).

### TRAIL-R4-mediated constitutive Akt activation in HeLa cells contributes to cell resistance to TRAIL-induced apoptosis and to increased cell proliferation

To explore the molecular basis of the deregulated death commitment and proliferation potential in HeLa cells expressing TRAIL-R4 ectopically, we next analyzed the activation status of Akt, a survival pathway that plays a central role in diverse cellular functions, including survival, growth, and proliferation [16].

**Figure 3:**
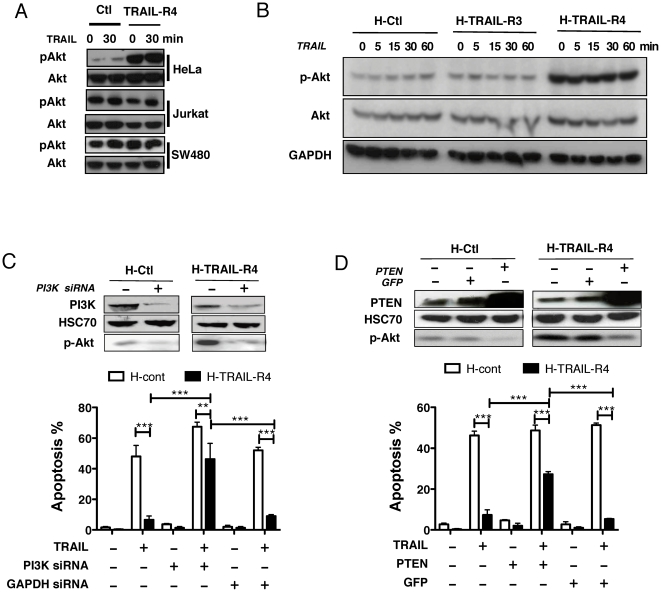
TRAIL-R4-mediated constitutive Akt activation contributes to cell resistance to TRAIL-induced apoptosis in HeLa cells. (A) Total Akt and phospho-Akt were monitored by western blot from HeLa, Jurkat and SW480 cells expressing TRAIL-R4 and compared to control cells (Ctl). Unstimulated or TRAIL stimulated (500 ng/ml for 30 minutes) cell samples are shown. (B) H-Ctl, H-TRAIL-R4 or HeLa cells expressing TRAIL-R3 (H-TRAIL-R3) were stimulated with TRAIL (500 ng/ml) for the indicated time and total Akt or phospho-Akt expression was assessed as above. Sample loading was assessed using GAPDH for normalization. (C) H-Ctl and H-TRAIL-R4 cells were transfected with a siRNA targeting PI3K or a scramble siRNA for 48 hours and stimulated or not with TRAIL (500 ng/ml) for 6 hours. Phosphorylation of Akt and PI3K expression levels were analyzed by western blot, and apoptosis was analyzed by Hoechst staining. (D) H-Ctl and H-TRAIL-R4 cells were transfected with a GFP mock vector or with a vector encoding PTEN for 24 hours. Expression levels of phospho-Akt and PTEN were analyzed by western blot and apoptosis induced by TRAIL was monitored as above. **P<0.01 and ***P<0.001, one-way ANOVA with Bonferroni's multiple comparison test.

**Figure 4:**
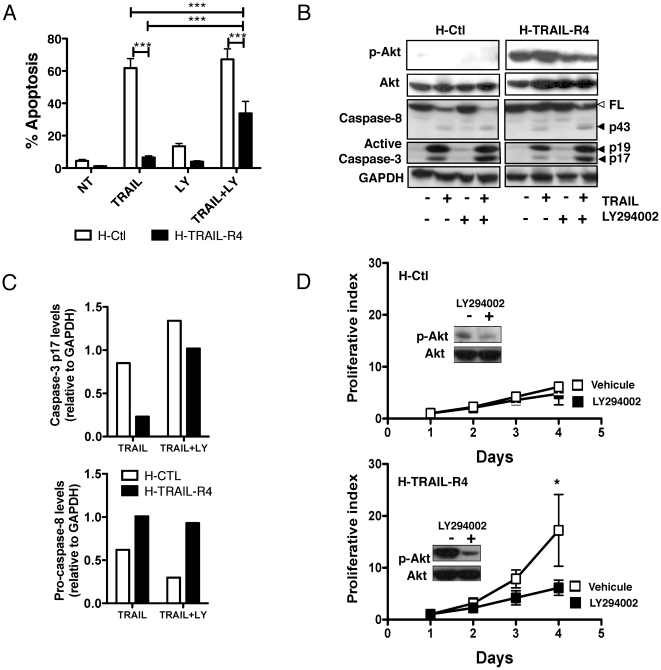
Inactivation of Akt restores partial sensitivity to TRAIL-induced cell death in TRAIL-R4 expressing cells and restores normal cell proliferation index. (A) HeLa H-Ctl and H-TRAIL-R4 cells were left untreated or pretreated for 1 hour with the Akt inhibitor LY294002 (100 µM) then stimulated or not with TRAIL (500 ng/ml) for 24 hours and apoptosis was analyzed after Hoechst staining. These results are representative of at least 3 independent experiments. Mean percentage of apoptotic cells and SD are shown (mean ± SD). ***P<0.001, one-way ANOVA with Bonferroni's multiple comparison test. (B) Cells were stimulated either with LY294002 (100 µM) then treated or not with TRAIL as above and cell lysates were processed by western blot for the analysis of Akt phosphorylation, caspase-8 and active caspase-3. Filled arrows show caspase cleavage products. The empty arrow shows full length caspase-8. Densitometry analysis of caspase-3 p17 and caspase-8 full-length immunoreactive bands were obtained using ImageJ software, normalized with respect to GAPDH, and plotted in (C). (D) Cellular growth in the presence or the absence of Akt inhibitor LY294002 (10 µM) was monitored during 4 days by flow cytometry analysis using CFSE. LY294002 was applied every day to the cell culture supernatant to afford sustained inhibition of Akt.

Strikingly, while Akt appeared to be constitutively activated in Jurkat and SW480 cells irrespective of TRAIL-R4 ectopic expression, a differential Akt phosphorylation profile was detected in H-TRAIL-R4 cells as compared to control parental HeLa cells (Figure 3A). Constitutive activation of Akt in HeLa cells was TRAIL-independent as TRAIL stimulation only marginally induced Akt phosphorylation in H-Ctl cells (Figure 3B). Activation of Akt in these cells appeared to be restricted to TRAIL-R4. Accordingly, ectopic expression of TRAIL-R3 (Figure 3B), TRAIL-R2 (not shown) or a chimeric TRAIL receptor encoding the extracellular domain of TRAIL-R1 fused to TRAIL-R2 (Figure S2) induced no change in Akt activation, contrary to a chimeric construct encoding TRAIL-R4 intracellular domain (Figure S2). Inhibition of Akt phosphorylation either using siRNA targeting the regulatory subunit of PI3K (Figure 3C), or by over-expressing PTEN (Figure 3D) sensitized H-TRAIL-R4 cells to TRAIL-induced cell death, indicating that cell resistance to TRAIL in these cells is, at least partly, due to the sustained activation of the Akt pathway. Accordingly, the pharmacological inhibitor of Akt phosphorylation, LY294002, significantly restored TRAIL-induced apoptosis in H-TRAIL-R4 cells (Figure 4A). Notably, sensitization to TRAIL-induced cell death was not associated with an increase in caspase-8 cleavage, but rather with an increase in caspase-3 processing (Figure 4B and C), suggesting that Akt-mediated inhibition most likely occurs downstream of the TRAIL DISC probably at the mitochondrial or post mitochondrial level. Remarkably, LY294002 reduced H-TRAIL-R4-mediated cell proliferation to levels comparable to those of control parental cells (Figure 4D).

Altogether our findings suggest that TRAIL-R4 in HeLa cells is a regulatory receptor whose anti-apoptotic functions involve both TRAIL DISC targeting and activation of the Akt survival pathway.

## Discussion

TRAIL is an attractive anti-tumoral agent owing to its ability to selectively induce apoptosis in tumor cells [17], [18] and thus TRAIL derivatives or recombinant TRAIL preparations have entered clinical trials [1]. The molecular mechanisms governing TRAIL-induced cell death or signal transduction remain, however, only partially understood. Cell resistance to TRAIL-induced cell death can arise both from the inhibition of the apoptotic machinery, or more specifically from the deregulation of the expression and/or the functionality of TRAIL receptors. Likewise, loss of TRAIL-R1 or TRAIL-R2 expression [19], [20], [21], [22] or expression of TRAIL-R3 or TRAIL-R4, two main antagonistic receptors [9], [10], [11], [12], [13], abrogate TRAIL-induced cell death selectively, without affecting the apoptotic machinery triggered by other members of the TNF family, nor the intrinsic pathway. Likewise, overexpression of TRAIL-R4 protects tumor cells against TRAIL-induced cell death by regulating caspase-8 activation at the DISC level [10], [15].

**Figure 5:**
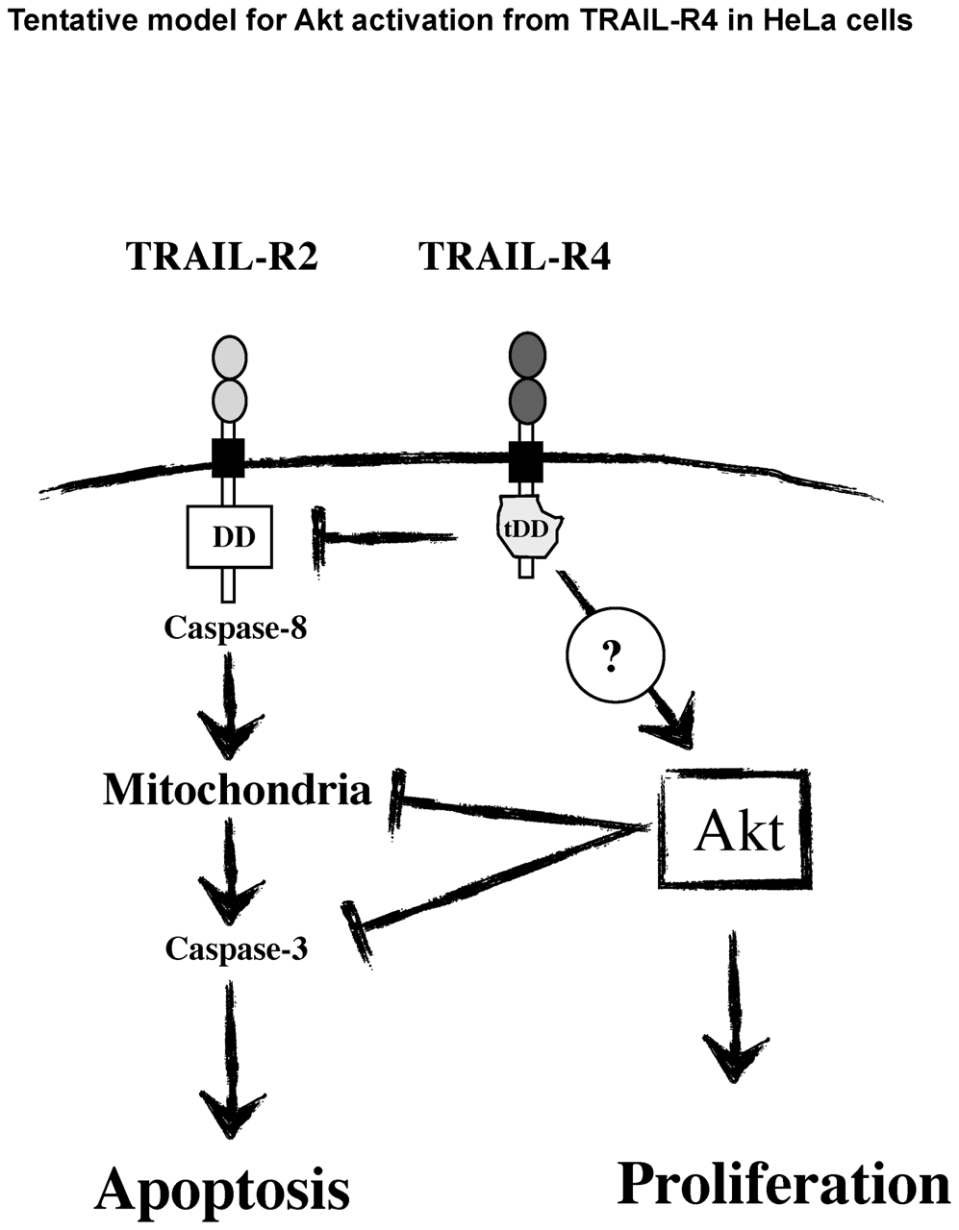
Model for Akt activation from TRAIL-R4 in HeLa cells. TRAIL-R4 is known to inhibit caspase-8 activation induced by TRAIL-R2 upon TRAIL stimulation. We propose here that TRAIL-R4 may, in addition, trigger Akt activation in a ligand independent manner. Constitutive activation of Akt in HeLa cells would therefore inhibit caspase-3 activation at the mitochondrial or post-mitochondrial level to inhibit apoptosis induced by TRAIL, and could possibly foster cell proliferation.

In the present study, we provide evidence that TRAIL-R4 in HeLa cells a) partially protects from TRAIL-induced apoptosis and b) enhances cell proliferation index through Akt activation in a TRAIL-independent manner. Remarkably, TRAIL-R4-mediated Akt activation in HeLa cells leads to drastic morphological changes, such as reduced cell size and loss of adhesion, suggesting that TRAIL-R4 may exhibit pro-metastatic properties (Figure 5). These findings, however, may be restricted to tumor cell lines expressing low levels of active Akt, since contrary to HeLa cells, no morphological or proliferative changes were detected in Jurkat and SW480 cells, which express high levels of active Akt. Our findings nonetheless clearly suggest, for the first time, that TRAIL-R4 exhibits unexpected signaling properties conferring cellular growth advantage both *in vitro* and *in vivo.*

Besides apoptosis triggering, and depending on the cell line or type, TRAIL stimulation can induce non apoptotic signaling pathways including Akt [23], [24], [25], [26], NF-kB [27] or MAPK [28], [29], cell proliferation [26], [30], [31] or differentiation [32], [33], [34]. It has recently been proposed that the activation of non apoptotic signaling pathways by TRAIL involve the formation of a cytosolic secondary complex which, contrary to TNF [35], allows the recruitment of adaptor proteins and kinases including TRAF2, RIP or NEMO [36]. Activation of Akt by TRAIL-R4 in HeLa cells by such a secondary complex is unlikely, as this pathway appears to be activated in a TRAIL-ligand independent manner. Moreover, we have not yet been able to identify this secondary complex (not shown).

How TRAIL-R4 mediates Akt activation in HeLa cells remains unknown for the moment. TRAIL-R4 shares relatively high homology with TRAIL-R1 and TRAIL-R2 [7] and is the only TRAIL antagonistic receptor that harbors an intracellular domain. Interestingly, ectopic expression of the TRAIL-R4 intracellular domain in HeLa cells induces constitutive AKT activation. This domain would therefore provide an ideal docking site for a putative binding partner. It is therefore tempting to speculate that the intracellular domain of TRAIL-R4 may interact with membrane associated or cytosolic proteins already known to bind to TRAIL-R1 or TRAIL-R2 such as GSK3 [37], ARAP1 [38], DAP3 [39], Burton's tyrosine kinase [40], or PRTM5 [41]. The exact molecular mechanism involved in this regulation therefore needs to be further investigated.

Altogether, our findings indicate for the first time that, besides inhibiting TRAIL-induced cell death, TRAIL-R4 is able to regulate the PI3K/Akt signaling pathway in cell type-dependent manner leading to cell resistance to apoptosis and to enhanced cellular growth.

## Materials and Methods

### Reagents and antibodies

His-tagged recombinant soluble human TRAIL and Fc-TRAIL-R2 were produced and used as described previously [42]. For Western blotting experiments, anti-GAPDH, anti-PI3K, anti-HSC70 were obtained from Santa Cruz Biotechnology (CA, USA), anti-phospho-Akt from Upstate (Milipore, Molsheim, France), anti-Akt, and anti-PTEN from Cell Signaling (Ozyme, Saint Quentin Yvelines, France). Antibodies used for flow cytometry, anti-TRAIL-R1 (wB-K32), anti-TRAIL-R2 (B-L27), anti-TRAIL-R3 (wB-B44), anti-TRAIL-R4 (wB-P30), and anti-TRAIL were from Diaclone (Besançon, France). The PI3K/AKt inhibitor LY294002 was purchased from Cell Signaling (Ozyme, Saint Quentin Yvelines, France).

### Cell culture and transfection

HeLa (human cervix carcinoma), SW480 (Colon carcinoma) and Jurkat cells (T Lymphoma) were obtained from the ATCC. HeLa and SW480 cells were cultured in high-glucose Dulbecco's modified Eagle's medium (Sigma-Aldrich, Lyon, France) supplemented with 10% fetal calf serum (Gibco-BRL, Erigny, France), and Jurkat cells were cultured in RMPI as above. Transfection of HeLa cells was carried out using JetsiENDO™ reagent (Eurogentec, Angers, France), according to the manufacturer's instructions using the following PI3K siRNA (5′-GGGUGUGGAUUACACCAUU-3′) and siRNA control (Invitrogen, Cergy Pontoise, France). PTEN transfections were performed with the pSG5L-HA-PTEN construct (kindly provided by Dr William Sellers, Dana-Farber Cancer Institute, Boston) using TransPEI™ (Eurogentec).

### Retroviral production and cell transduction

The retroviral vector pMSCVpuro and the generation of viruses have previously been described (30). TRAIL-R4 full-length construct was subcloned from a pCR-3 vector (Invitrogen) to the retroviral vector pMSCV-puro as a HindII-XhoI fragment. HeLa cells were transduced for 16 hours with viral supernatants containing polybrene (8 µg/ml), washed in phosphate buffered saline (PBS), and selected in complete medium containing puromycin (2.5 µg/ml).

### Measurement of cell viability and apoptosis

Cell viability assays were performed in 96-well plates. 10^4^ cells per well were incubated at 37°C for 24 hours with increasing concentrations of His-TRAIL (from 0 to 10,000 ng/ml). Cell viability was determined by the PMS/MTS method, according to the manufacturer's specifications (Promega, Madison, WI, USA.). Apoptosis was assessed by Hoechst staining by determining the percentage of condensed nuclei from at least 300 cells per condition. For Akt inhibition, cells were pretreated with LY294002 (100 µM) one hour before TRAIL treatment (500 ng/ml, 5 hours). PI3K inhibition and PTEN expression were measured 48 and 24 hours hours after transfection respectively and cells were simultaneously treated 5 hours with 500 ng/ml of His-TRAIL.

### Cell proliferation

To measure cell proliferation the different cellular populations were plated at the same density and counted each day. CellTrace™ CFSE Cell Proliferation Kit (Molecular Probes, Invitrogen) was used to measure cell proliferation using a LSRII flow cytometer (DB Biosciences) and the ModFIT Software (Verity Software House Topsham, ME) was applied to determine the proliferation index.

### Western blotting

Lysates were resolved by sodium dodecyl sulfate- polyacrylamide gel electrophoresis and transferred to nitrocellulose membranes. Nonspecific binding sites were blocked in PBS containing 0.05% Tween 20 and 5% powdered milk. Immunoblots were then incubated with specific primary antibody, followed by horseradish peroxidase-conjugated secondary antibody, and developed by the enhanced chemiluminescence method according to the manufacturer's protocol (Pierce, Rockford, IL).

### *In vivo* studies

Six weeks old female athymic nude mice (Harlan, Le Malcourle, Gannat) were subcutaneously xenografted with 1×10^6^ H-Ctl in the right flank and 1×10^6^ H-TRAIL-R4 in the left flank (n = 10). Tumor volume was obtained after caliper measurement of the tumor and the formula (l×l×L)/2 with l the smaller and L the higher dimension.

## Supporting Information

Figure S1 (**A**) The proliferative index of H-Ctl and H-TRAIL-R4 cells was measured in the presence or in the absence of 10 µg recombinant Fc-TRAIL-R2, as described in the manuscript Figure 1E. Fc-TRAIL-R2 was added to the culture daily for 4 days. (B) Representative picture of nude mice xenografted with HeLa control (H-Ctl on the left flank) and HeLa expressing TRAIL-R4 (H-TRAIL-R4 on the right flank) and the corresponding tumors harvested from mice pictured.
(TIFF)

Figure S2 (**A**) Schematic representation of TRAIL receptor chimeric constructs (OM043, OM050 and OM051). Vectors were constructed using standard cloning procedures. TRAIL-R2 and TRAIL-R4 intracellular domains (icd) were obtained by polymerase chain reaction from pCRIII vectors encoding full length TRAIL-R2 and TRAIL-R4 as described earlier [10], with the following primer pairs: TRAIL-R2 forward primer (5′- GTC GAC TGT TCT CTC TCA GGC ATC-3′); reverse primer (5′- CTC GAG CGG CCG CCA GTG TGA TGG-3′) and TRAIL-R4 forward primer (5′- GTC GAC TAT CAC TAC CTT ATC ATC -3′); reverse primer (5′- CTC GAG TCA CAG GCA GGA CGT AGC -3′) containing a SalI and a XhoI site. Oligonucleotide primers and Pfu polymerase were purchased from Eurogentech (Angers, France) and Sigma-Aldrich (Lyon, France) respectively. The resulting amplified fragments were subcloned into pCR-Blunt (Invitrogen, Cergy Pontoise, France) and checked by sequencing. TRAIL-R2-icd and TRAIL-R4-icd were subcloned between the SalI and XhoI sites of pCRIII vectors encoding the extracellular domains (ecd) of TRAIL-R1 (aa 1–239, PS688), TRAIL-R2 (aa 1–212, PS664) or TRAIL-R4 (aa1–211, PS690), kindly provided by Dr Pascal Schneider (Lausanne, Switzerland). Resulting TRAIL-R1ecd-TRAIL-R2icd, TRAIL-R4ecd-TRAIL-R2icd and TRAIL-R2ecd-TRAIL-R4icd DNA fragments were subcloned into a pMSCV-Puro retroviral vector between HindIII and XhoI, generating pMSCV-Puro-TRAIL-R1ecd-TRAIL-R2icd (OM043), pMSCV-Puro-TRAIL-R4ecd-TRAIL-R2icd (OM051) and pMSCV-Puro-TRAIL-R2ecd-TRAIL-R4icd (OM050). (**B**) Receptor expression in HeLa cells was analyzed by flow cytometry after infection of TRAIL receptor fusion constructs (OM043, OM050 and OM051) or the corresponding empty vector (OM181). (**C**) Biochemical analysis of TRAIL chimeric receptors. Immunoprecipitations were performed using a control antibody (lanes 1, 4, 7, 10, 13 and 16) or antibodies targeting TRAIL-R1 (lanes 2, 3, 5 and 6), TRAIL-R2 (lanes 8, 9, 11 and 12) and TRAIL-R4 (lanes 14, 15, 17 and 18) from DIACLONE (Besançon, France). Antibodies were applied either after lysis (1, 3, 4, 6, 7, 9, 10, 12, 13, 15, 16 and 18), or on intact cells (2, 5, 8, 11, 14 and 17) for 1 h on ice. The latter samples were subsequently subjected to lysis in NP40 lysis buffer and samples were precleared on sepharose 6B beads before immunoprecipitation using agarose protein G beads. Samples were then washed extensively and subjected to western blot using anti-TRAIL-R1, -R2 and -R4 antibodies from Milipore (Molsheim, France). Note that TRAIL-R4's extracellular domain is not recognized by the anti-TRAIL-R4 antibody from Chemicon. *ns stands for non specific. (**D**) AKT activation was analyzed by western blot as described in the text.
(TIFF)
