# Supplementary figures and images for: TRAIL-R4 Promotes Tumor Growth and Resistance to Apoptosis in Cervical Carcinoma HeLa Cells through AKT
Source: PLoS One. 2011 May 20;6(5):e19679. doi: 10.1371/journal.pone.0019679 (PMC3098831; doi:10.1371/journal.pone.0019679)

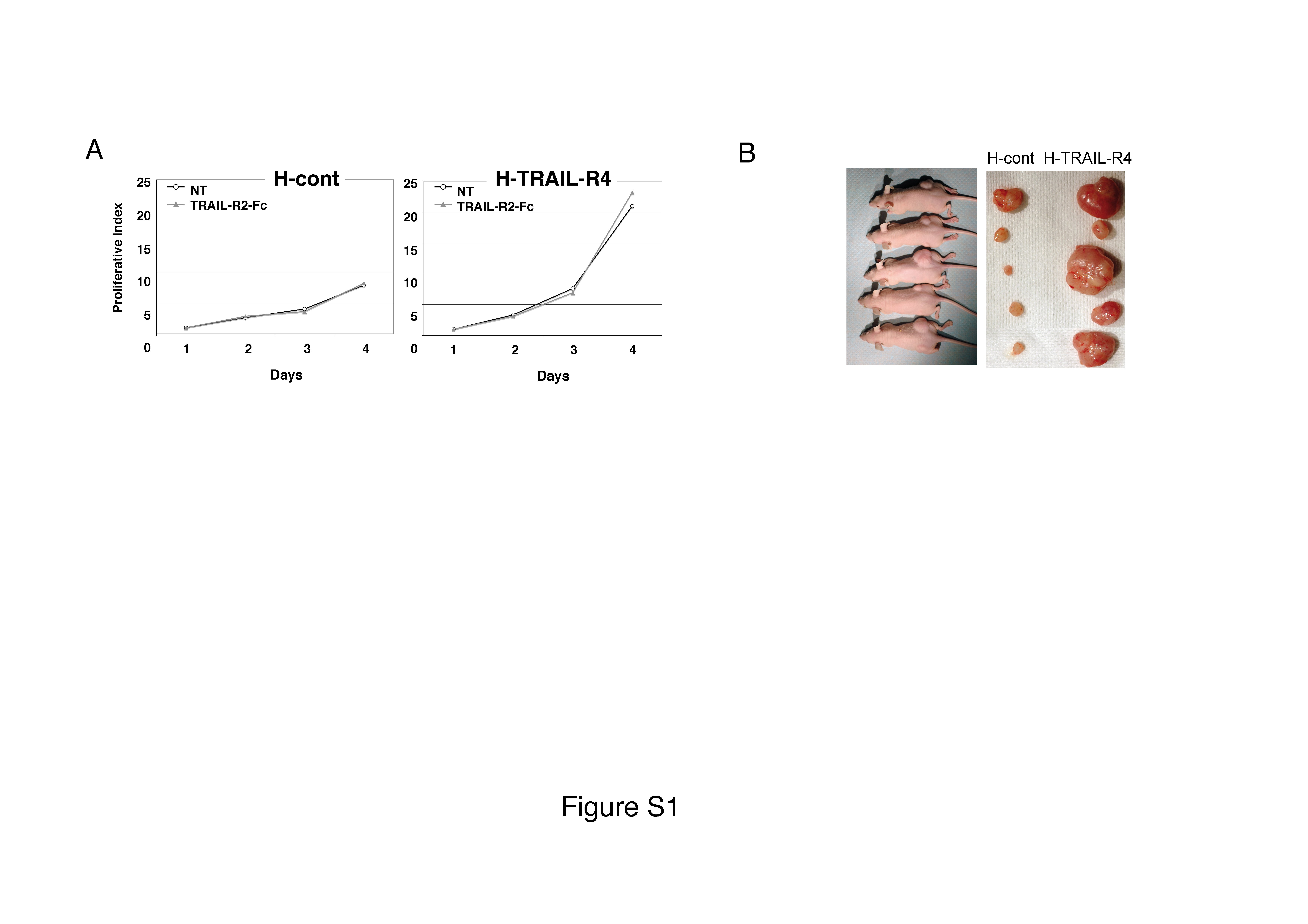

Supplement: Figure S1 — (A) The proliferative index of H-Ctl and H-TRAIL-R4 cells was measured in the presence or in the absence of 10 µg recombinant Fc-TRAIL-R2, as described in the manuscript Figure 1E. Fc-TRAIL-R2 was added to the culture daily for 4 days. (B) Representative picture of nude mice xenografted with HeLa control (H-Ctl on the left flank) and HeLa expressing TRAIL-R4 (H-TRAIL-R4 on the right flank) and the corresponding tumors harvested from mice pictured. (TIFF) [file pone.0019679.s001.tiff]

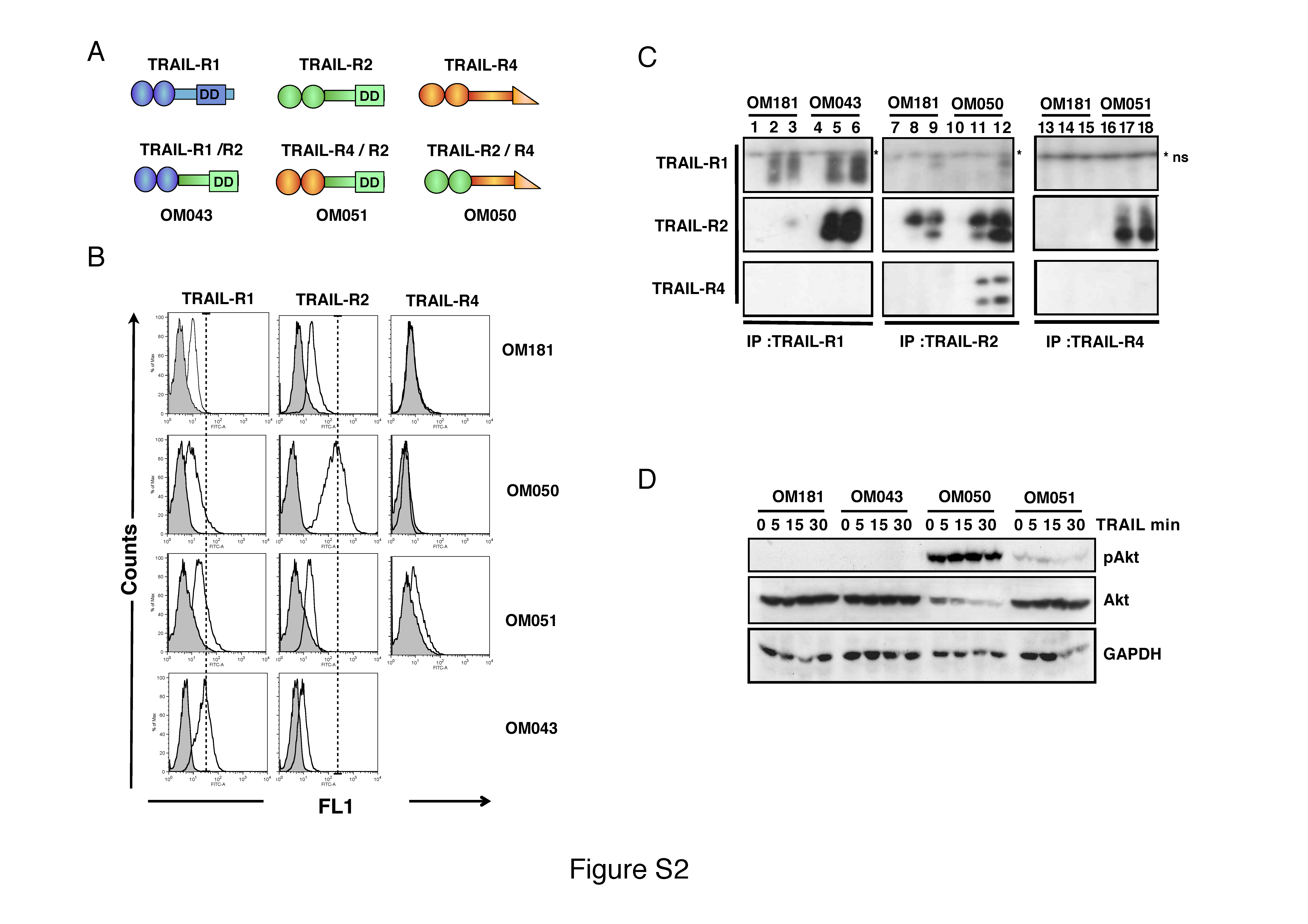

Supplement: Figure S2 — (A) Schematic representation of TRAIL receptor chimeric constructs (OM043, OM050 and OM051). Vectors were constructed using standard cloning procedures. TRAIL-R2 and TRAIL-R4 intracellular domains (icd) were obtained by polymerase chain reaction from pCRIII vectors encoding full length TRAIL-R2 and TRAIL-R4 as described earlier [10], with the following primer pairs: TRAIL-R2 forward primer (5′- GTC GAC TGT TCT CTC TCA GGC ATC-3′); reverse primer (5′- CTC GAG CGG CCG CCA GTG TGA TGG-3′) and TRAIL-R4 forward primer (5′- GTC GAC TAT CAC TAC CTT ATC ATC -3′); reverse primer (5′- CTC GAG TCA CAG GCA GGA CGT AGC -3′) containing a SalI and a XhoI site. Oligonucleotide primers and Pfu polymerase were purchased from Eurogentech (Angers, France) and Sigma-Aldrich (Lyon, France) respectively. The resulting amplified fragments were subcloned into pCR-Blunt (Invitrogen, Cergy Pontoise, France) and checked by sequencing. TRAIL-R2-icd and TRAIL-R4-icd were subcloned between the SalI and XhoI sites of pCRIII vectors encoding the extracellular domains (ecd) of TRAIL-R1 (aa 1–239, PS688), TRAIL-R2 (aa 1–212, PS664) or TRAIL-R4 (aa1–211, PS690), kindly provided by Dr Pascal Schneider (Lausanne, Switzerland). Resulting TRAIL-R1ecd-TRAIL-R2icd, TRAIL-R4ecd-TRAIL-R2icd and TRAIL-R2ecd-TRAIL-R4icd DNA fragments were subcloned into a pMSCV-Puro retroviral vector between HindIII and XhoI, generating pMSCV-Puro-TRAIL-R1ecd-TRAIL-R2icd (OM043), pMSCV-Puro-TRAIL-R4ecd-TRAIL-R2icd (OM051) and pMSCV-Puro-TRAIL-R2ecd-TRAIL-R4icd (OM050). (B) Receptor expression in HeLa cells was analyzed by flow cytometry after infection of TRAIL receptor fusion constructs (OM043, OM050 and OM051) or the corresponding empty vector (OM181). (C) Biochemical analysis of TRAIL chimeric receptors. Immunoprecipitations were performed using a control antibody (lanes 1, 4, 7, 10, 13 and 16) or antibodies targeting TRAIL-R1 (lanes 2, 3, 5 and 6), TRAIL-R2 (lanes 8, 9, 11 and 12) and TRAIL-R4 (lanes 14, 15, 17 and 18) from [file pone.0019679.s002.tiff]
